# Transcriptomic profile of the mice aging lung is associated with inflammation and apoptosis as important pathways

**DOI:** 10.18632/aging.203039

**Published:** 2021-05-12

**Authors:** Jazmin Calyeca, Yalbi I. Balderas-Martínez, Moisés Selman, Annie Pardo

**Affiliations:** 1 Division of Pulmonary Allergy and Critical Care Medicine, Aging Institute, Department of Medicine, University of Pittsburgh, Pittsburgh, Pennsylvania 15219, USA; 2 Division of Pulmonary, Critical Care and Sleep Medicine, Department of Internal Medicine, Davis Heart and Lung Research Institute, Ohio State University, Columbus, Ohio 43210, USA; 3 Instituto Nacional de Enfermedades Respiratorias “Ismael Cosío Villegas”, Ciudad de México 14080, México; 4 Facultad de Ciencias, Universidad Nacional Autónoma de México, Ciudad de México 04510, México

**Keywords:** aging lung, senescence, inflammaging, lung fibrosis

## Abstract

Aging is a universal biological process characterized by a progressive deterioration in functional capacity and an increased risk of morbidity and mortality over time. In the lungs, there are considerable changes in lung structure and function with advancing age; however, research on the transcriptomic profile implicated in this process is scanty. In this study, we addressed the lung transcriptome changes during aging, through a global gene expression analysis of normal lungs of mice aged 4- and 18-months old. Functional pathway enrichment analysis by Ingenuity Pathway Analysis (IPA) revealed that the most enriched signaling pathways in aged mice lungs are involved in the regulation of cell apoptosis, senescence, development, oxidative stress, and inflammation. We also found 25 miRNAs significantly different in the lungs of old mice compared with their younger littermates, eight of them upregulated and 17 downregulated. Using the miRNet database we identified TNFα, mTOR, TGFβ, WNT, FoxO, Apoptosis, Cell cycle, and p53 signaling pathways as the potential targets of several of the dysregulated miRNAs supporting that old lungs have increased susceptibility for apoptosis, inflammation, and fibrosis. These findings reveal differential expression profiles of genes and miRNAs affecting cell survival and the inflammatory response during lung aging.

## INTRODUCTION

Aging represents a biological process characterized by a decline in functional capacity and an increased risk of disease and mortality. The cellular and molecular changes associated with aging are partially understood; still, they involve among others, genome instability, telomere attrition, epigenetic alterations, loss of proteostasis, deregulated nutrient-sensing, mitochondrial dysfunction, cellular senescence, depletion of adult stem cell reservoirs, and altered cellular communication [1]. All these processes result in a progressive decrease in tissue-repair capacity and increased morbidity and mortality.

In the lungs, these changes may result in a progressive functional and structural impairment, which may promote the development of multiple disease processes [2, 3]. Even in healthy individuals, the decline of lung function starts early in life [3, 4]. Structural changes with age include a progressive loss of alveolar surface area and enlargement of alveolar spaces with loss of lung elasticity resulting in the premature closing of the small airways.

Besides, exposure to environmental factors, such as chronic exposure to air pollution, dust, particulates, and gases, may contribute to accelerate aging of the lungs.

Up to date, however, the mechanisms associated with physiological and structural lung aging changes remain uncertain.

In a previous study, performed to clarify the role of aging in the development of lung fibrosis in Zmpste24 deficient mice that represent an accelerated aging-model, we evaluated by RNA microarray analysis differentially expressed genes in old compared to young WT mice lungs [5]. In the present study, we analyzed these data to identify different biological processes likely affected in physiological lung aging that would help to better understand the molecular changes associated with this process [5]. Additionally, we report here 25 miRNAs differentially expressed in the lungs of old WT mice compared with their young counterparts.

## RESULTS

**Figure 1:**
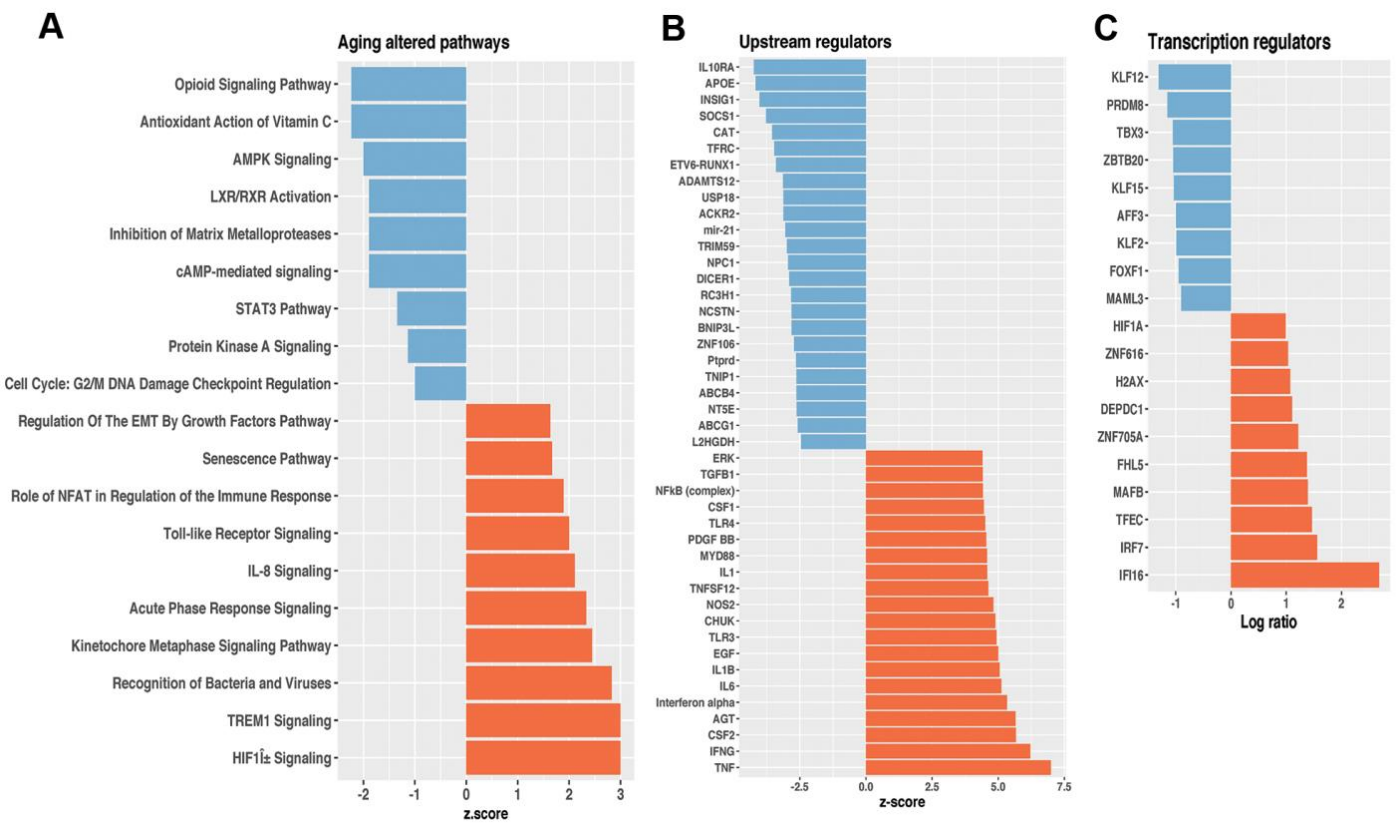
**Differentially expressed pathways and potential transcriptional regulators of lungs from old and young mice.** (**A**) Top 10 ranked high- and down Z-score pathways. (**B**) Upstream regulators. (**C**) Transcription regulators. Orange bars represent overexpressed genes; blue bars represent underexpressed genes, all of them at a significant level of p< 0.05. These results were obtained using Ingenuity Pathway Analysis™.

We first analyzed the transcriptional profile data of WT young and old mice lungs (Supplementary Table 1) and through Ingenuity Pathway Analysis™ (IPA), we identified over and under-represented biological functions, pathways, and potential transcriptional regulators within the 575 differentially expressed genes. Part of them were previously validated by qPCR (*Spp1, Timp1, Mmp8, Mmp12, Mmp13*, and *TFGbi*) [5] and three more (*Col1α1, Mmp14, Eln*) are shown in Supplementary Figure 1. The top 10 upregulated and 10 downregulated aging altered pathways (Supplementary Table 2) are shown in Figure 1A. The highest over-expressed pathway in old WT lungs compared with young ones was the triggering receptor expressed on myeloid cells-1 (TREM-1). Toll-like receptor signaling and p38 MAPK signaling also associated with inflammatory processes were highly upregulated. Likewise, acute phase response signaling, and recognition of bacteria and viruses were also over-expressed. Interestingly, senescence pathway, usually associated with aging, was also upregulated. By contrast, AMPK signaling, and Inhibition of Matrix Metalloproteases figured among downregulated pathways, and accordingly, the expression of the upstream regulator Insig1 was decreased in the lungs of old mice while the expression of the upstream regulators INF_ᵧ_, TNF, and TFG-β was increased (Figure 1B and Supplementary Table 3). Interestingly, in the nuclei, most of the differentially expressed molecules were transcription regulators. Using key Pathway Advisor-Clarivate and IPA, we found several transcription factors differentially expressed during lung aging. Upregulation included the sensor of the innate immune response interferon gamma inducible protein 16 (Ifi-16) and the hypoxia-inducible factor 1a (Hif1a), which was one of the most upregulated aging altered pathways (Figure 1A). Downregulated genes included the Kruppel-like factor 12 (KLF12), and Kruppel-like factor 15 (KLF15) showed in Figure 1C and Supplementary Table 4.

### Inflammation and apoptosis are major enrichment functional processes in the old lung

**Figure 2:**
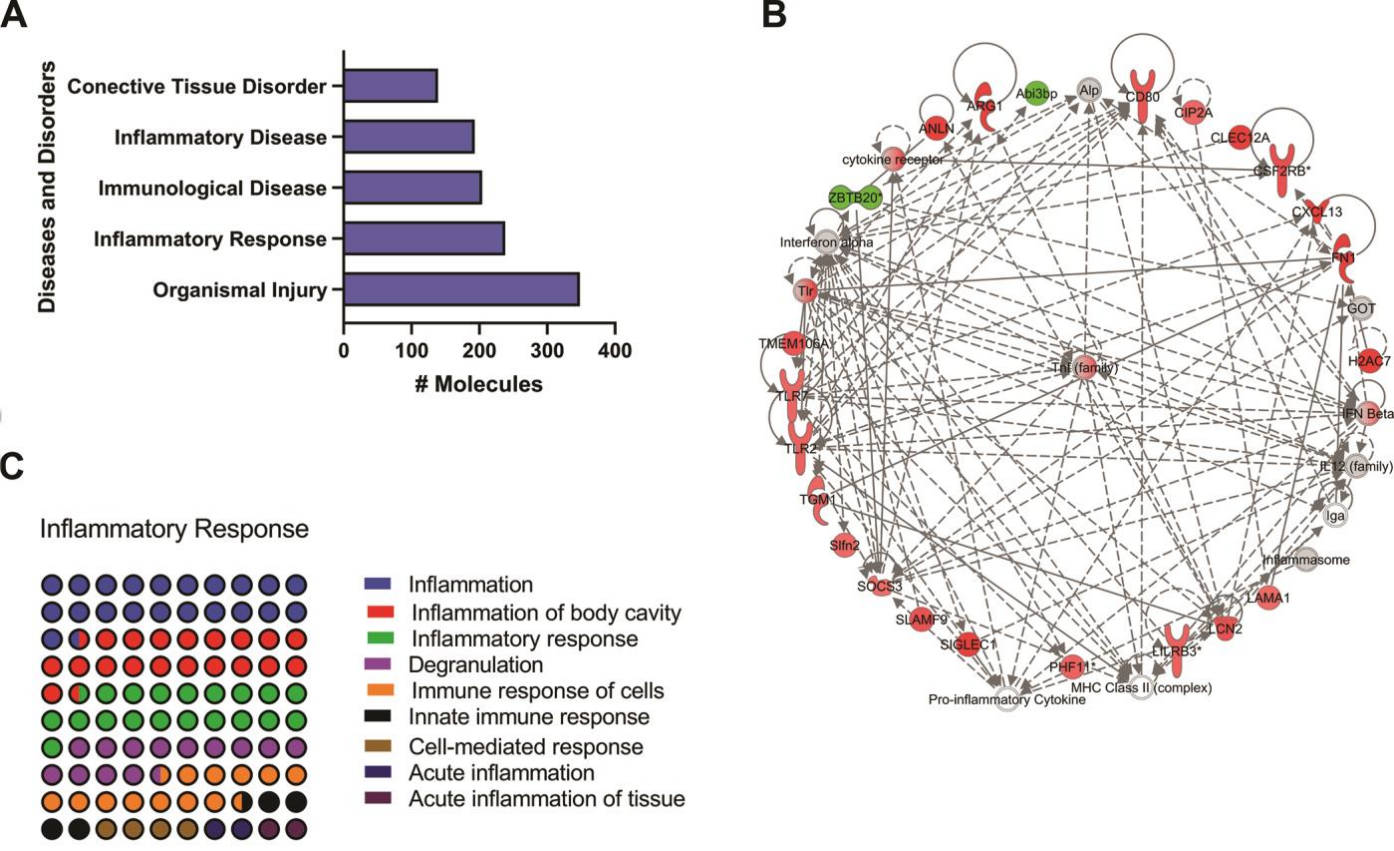
**Identification of top enriched diseases and disorders related pathways of old versus young mice lungs using IPA Z-score algorithm.** (**A**) Diseases and disorders category. (**B**) Percentage contribution of dysregulates pathways affecting the respiratory disease subcategory. (**C**) TNF interaction network. Colored nodes refer to genes found in our dataset (green downregulated; red upregulated). Uncolored nodes were not identified as differentially expressed in our experiment and were integrated into the computationally generated IPA networks.

Consistent with the revealed pathways, the top five functions from the diseases and disorders category revealed, among others, Inflammatory Response, Immunological disease, and Inflammatory disease as part of the five major enriched pathways (Figure 2A). The downstream processes involved, and the corresponding subcategories included inflammatory response, immune response of cells, and acute inflammation of tissue (Figure 2B). Further analysis in the networks implicated in the inflammatory response, demonstrated the activation of the multifunctional proinflammatory TNF interaction network as a major enriched pathway (Figure 2C).

**Figure 3:**
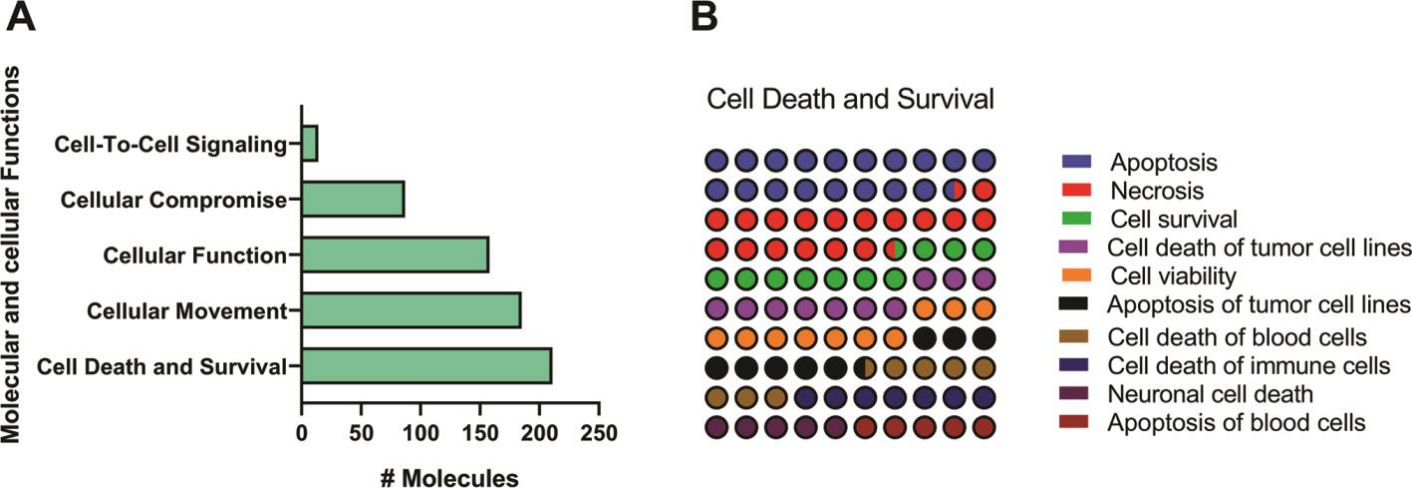
**Identification of top enriched molecular and cellular functions related pathways of old versus young mice lungs using IPA Z-score algorithm.** (**A**) Molecular and cellular functions category. (**B**) Percentage contribution of affected pathways in cell death and survival subcategory. Colored nodes refer to genes found in our dataset (green means downregulated; red means upregulated). Uncolored nodes were not identified as differentially expressed in our experiment and were integrated into the computationally generated IPA networks to indicate relevance to this network.

To further investigate functions associated with aging, we made an extended analysis of the top five functions from the molecular and cellular functions category (Figure 3A). This evaluation revealed Cell Death and Survival, and Cellular Movement, as the two major enriched pathways. We noticed that apoptosis of several lung cell populations was the more enriched subprocess (Figure 3B).

### Senescence signaling canonical pathway is enriched and activated during lung aging

**Figure 4:**
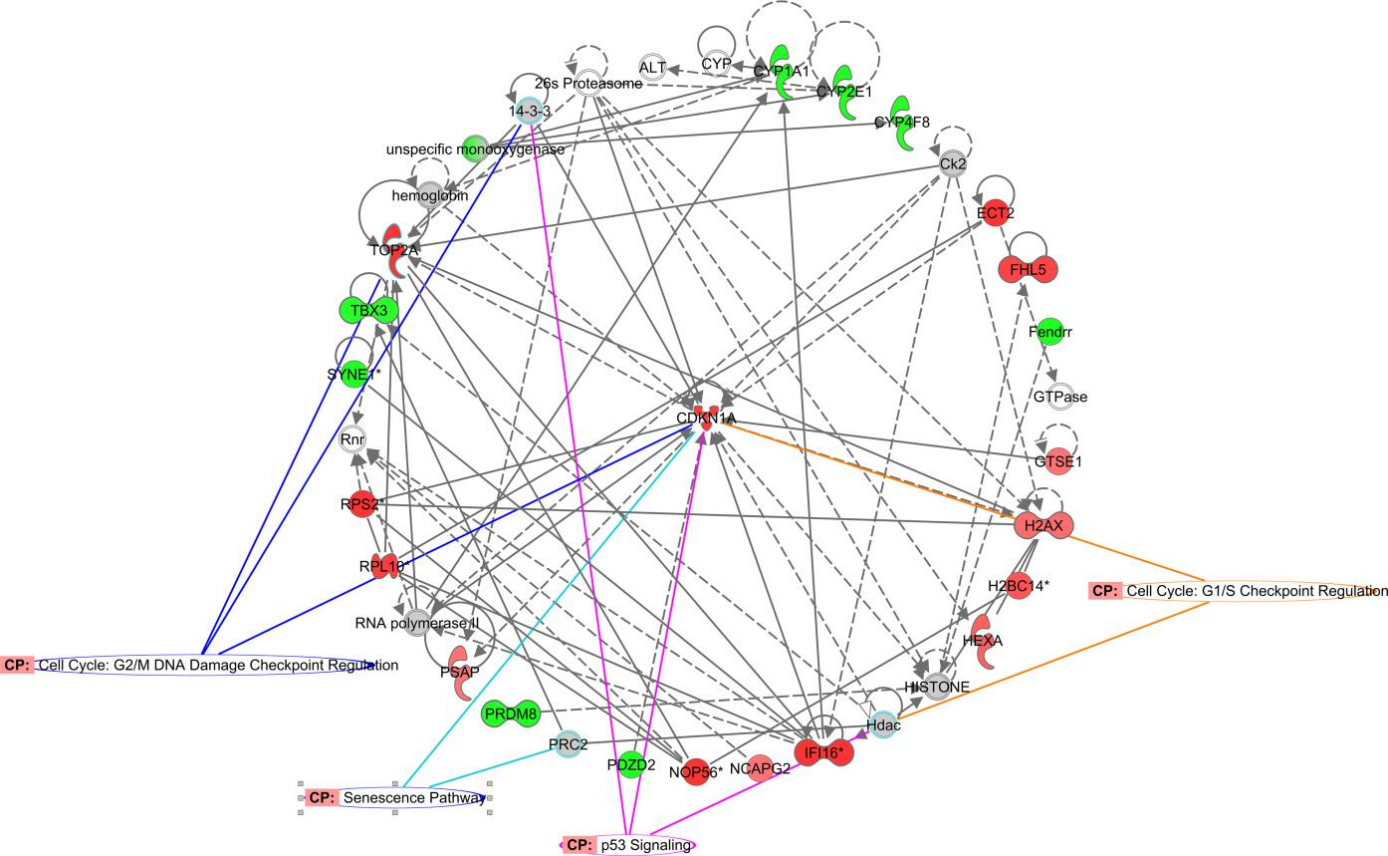
**CDKN1a functional network identified using Ingenuity Pathway Analysis™.** CDKN1a interaction network overlapping senescence associated pathways. Colored nodes refer to genes found in our dataset (green downregulated; red upregulated). Uncolored nodes were not identified as differentially expressed in our experiment and were integrated into the computationally generated IPA networks.

**Figure 5:**
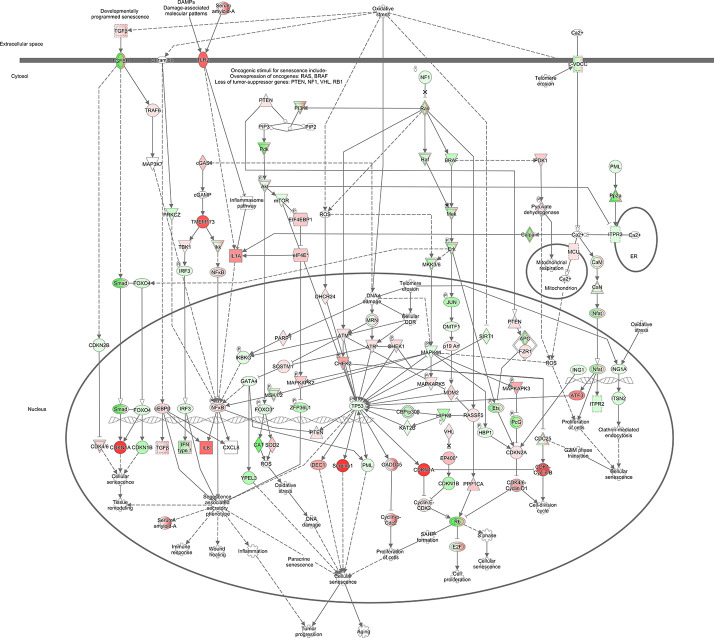
**Simulation of perturbations in senescence.** Senescence signaling perturbation pathway generated by using Molecular Activity Predictor (MAP), showing downstream consequences, and predicted inferred of senescence. Colored nodes refer to genes found in our dataset (green downregulated; red upregulated). Uncolored nodes were not identified as differentially expressed in our experiment and were integrated into the computationally generated IPA networks.

As shown in Figure 1A, senescence signaling also figured as one of the top 10 Z-score positive canonical enriched pathways. Accordingly, gene interaction networks showed CDKN1a (p21) as central gene related with cell cycle regulation and senescence (Figure 4). To further evaluate possible directional consequences, and inferred upstream regulator activity in senescence signaling, we used IPA tool Molecular Predicted Map on our data set to generate a general view of downstream consequences of senescence (Figure 5). Mechanistically, we found three major extracellular senescence inductors. One of them proposed that activation of developmental senescence by TGF-β can result in NFkβ-dependent IL-6 increased expression while increasing CDKN1a (p21) in a smad dependent manner. Supporting this, we found that both Damage Associated-Molecular Patterns (DAMPs) and Serum Amyloid-A promotes NFkβ-dependent IL-6 increased expression node mediated by TLR2/IL-1a pathway. Finally, high IL-6 expression is associated with Senescent-Associated Secretory Phenotype (SASP), cellular senescence, and aging.

**Figure 6:**
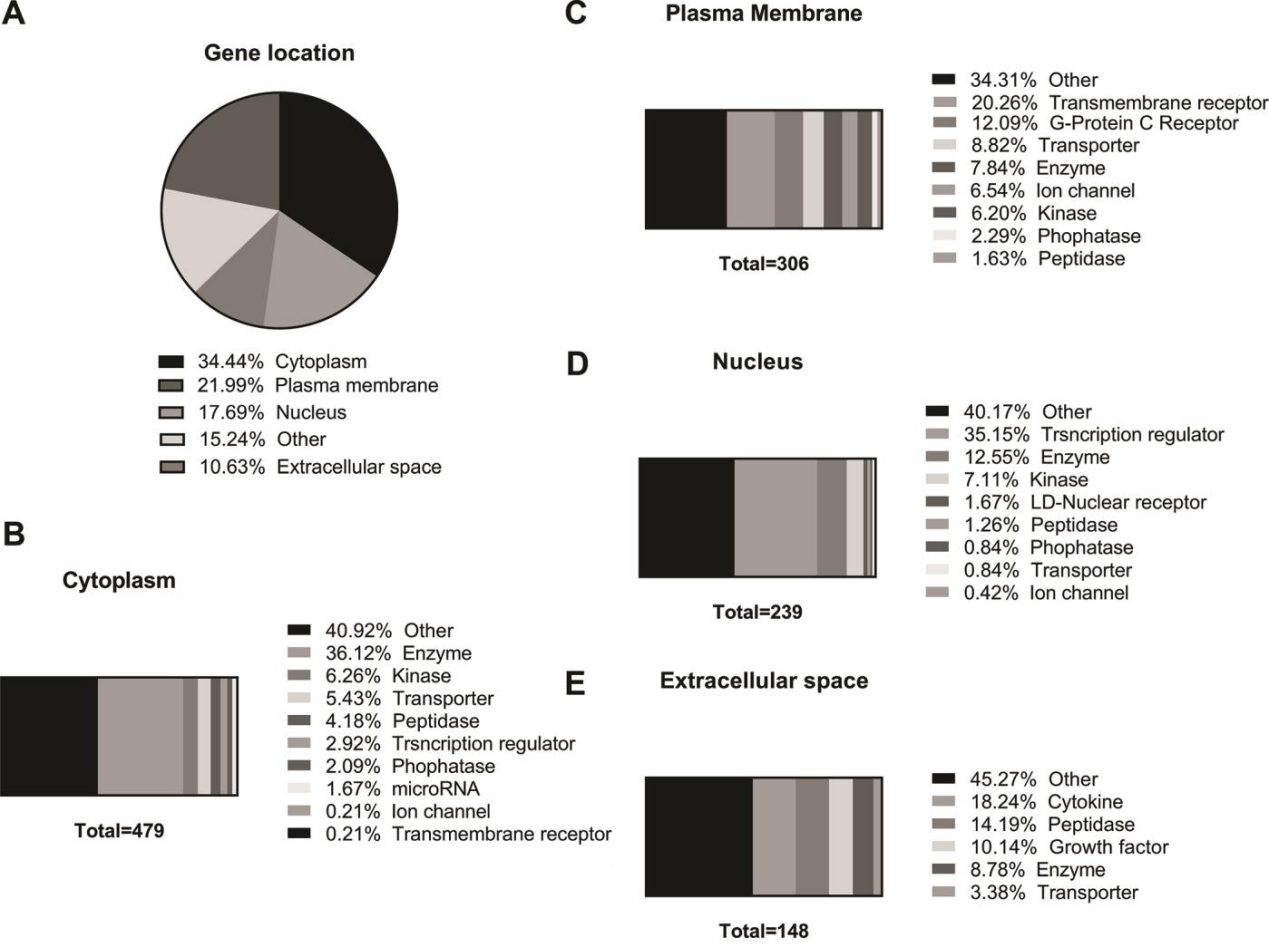
**Identification of predicted cellular localization of dysregulated genes in old versus young mice lungs using Ingenuity Pathway Analysis™.** (**A**) Pie graph of predicted cellular localization of the dysregulated 575 aged-changed genes, and their distinct molecular functions in (**B**) cytoplasm, (**C**) plasma membrane, (**D**) nucleus, and (**E**) extracellular space.

We also determined by IPA the cellular localization of the putative dysregulated proteins and their distinct molecular functions predicted from the 575 differentially expressed genes in the lung of old mice and which were the predicted aging-modulated cellular pathways (Supplementary Table 4). As shown in Figure 6A, the regulated proteins are distributed broadly in different parts of the cells, especially in the cytoplasm where one-third of the identified proteins were localized (Figure 6B). The differentially cytoplasmic expressed proteins were grouped into Enzyme, the highly identified expressed proteins, Kinase, Transporter, Peptidase, Phosphatase, Transporter Regulator, Ion channel, and Other (Figure 6B). Finally, around 20% of proteins were localized in the plasma membrane (Figure 6C), followed by the nucleus (Figure 6D) and the extracellular space (Figure 6E).

### Differentially expressed miRNAs during lung aging

**Figure 7:**
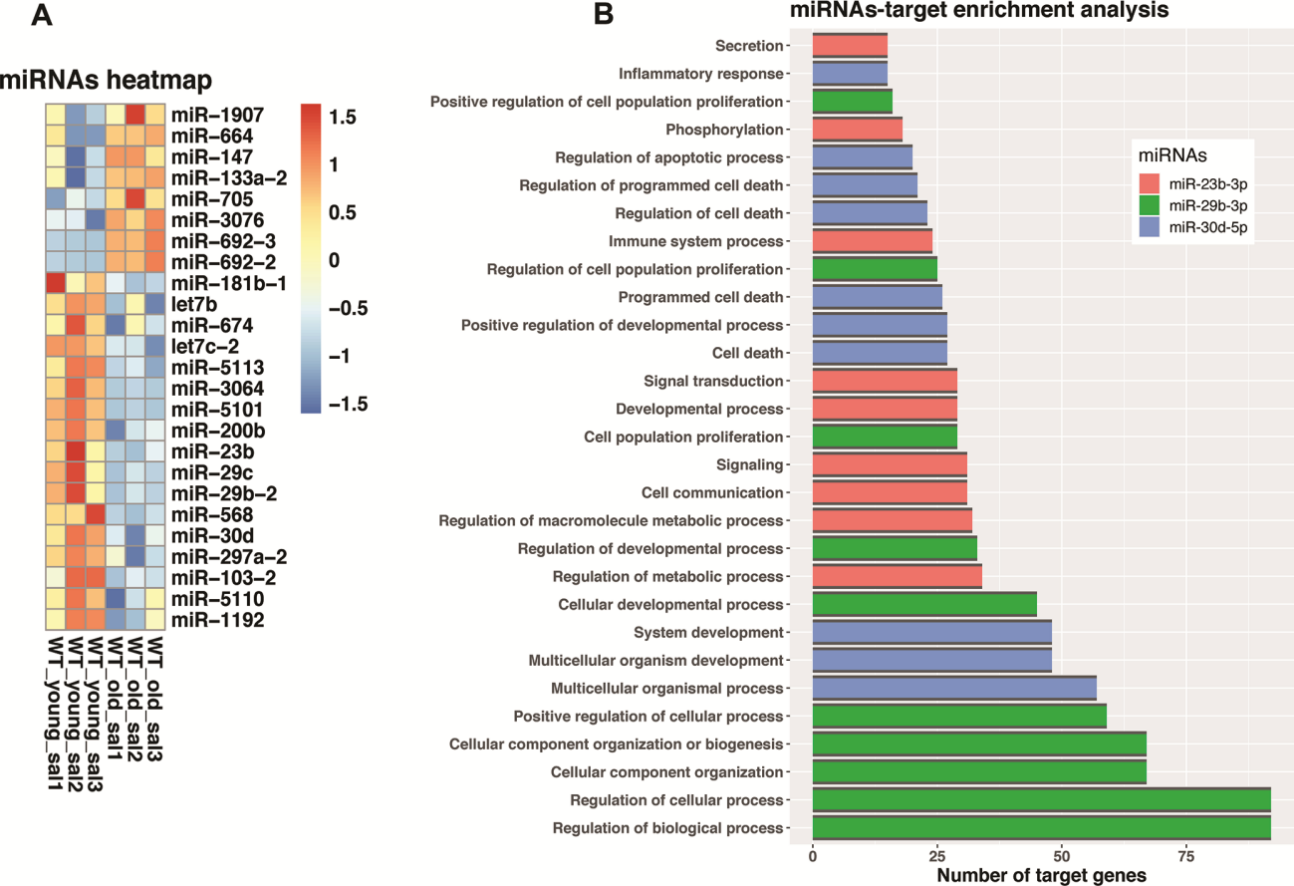
**Dysregulated miRNA expression and target genes in old versus young mice lungs.** (**A**) Changed expression levels of miRNAs. (**B**) miRNAs GSEA enrichment. The bar plot is showing GO biological processes that have an adjusted p-value < 0.5. Colors represent different miRNAs, miR-23b-3p (red), miR-29b-3p (green) and miR-30d-5p (blue).

Emerging evidence highlight the importance of miRNA-mediated regulation during aging. In this context, we investigate the potential involvement of miRNAs in the process of aging. We found that the expression of 25 miRNAs was significantly different in the lungs of old mice compared with their younger counterpart, eight of them were upregulated and 17 downregulated (Supplementary Table 5). A heatmap of the differentially expressed miRNAs is illustrated in Figure 7A. Interestingly, the most upregulated miRNAs were miRNA-664 and miRNA-147. Among other mRNAs and pathways, miRNA-664 target paired box protein 6 (PAX6) involved in some neoplastic diseases, and miRNA-147 that inhibits cell proliferation and regulates the ER-Stress-induced apoptosis [6–8]. In the interest of aging-lung associated diseases, miRNAs let7b, and miR200b, which belong to antifibrotic families of miRNAs, were found downregulated [9–11].

MiRNAs biological implications depends on their regulatory capacity. To predict functional implications of dysregulated miRNAs during lung aging, we determined miRNA-target interactions by constructing a miRNA-target gene regulatory network using miRNET v.2.0 database (Supplementary Figure 2 and Supplementary Table 6) [12]. Here, we found that several of our discovered dysregulated miRNAs were interconnected in the regulation of important biological processes, including inflammation, metabolism, and cell death, supporting the notion that apoptosis and the inflammatory response are critical pathways involved in lung aging. (Supplementary Table 7).

To gain more strength in our found miRNA-target interactions, we determine miRNA-dependent GO pathway regulation based on an enrichment analysis using Multimir bioinformatic tool base on their corresponding targets. (Supplementary Table 8). In accordance with this, we found that among all dysregulated miRNAs, during aging, miR-23-3p, miR-29b-3p, and miR-30d-5p targets were the most enriched (adj p-value < 0.05) (Figure 7B). Enriched pathways included the regulation of several important biological processes such as development and cell communication, and proliferation confirmed that regulation of inflammation and cell death are key processes of lung aging.

**Figure 8:**
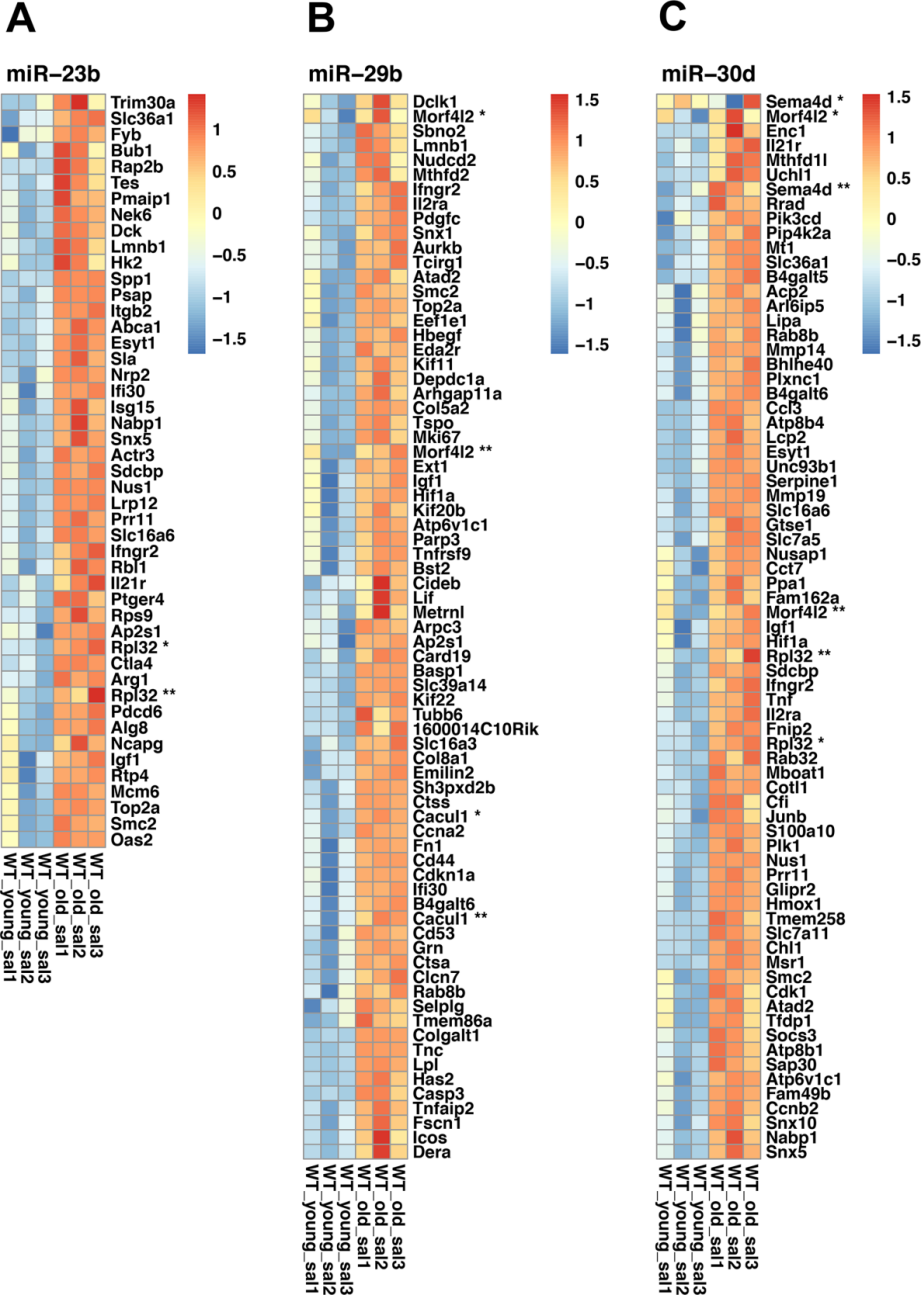
**Heatmaps of selected miRNAs.** (**A**) Heatmap of miR-23b differentially expressed target genes. (**B**) Heatmap of miR-29b differentially expressed target genes. (**C**) Heatmap of miR-30d differentially expressed target genes. Red indicates high expression, and blue, low expression. When duplicated, genes were marked with asterisks. Affymetrix probes IDs are shown inside the parenthesis. Rpl32 * (17469936), Rpl32 ** (17549432), Cacul1 * (17366081), Cacul (17548127), Morf4l2 * (17544754), Morrf4l2 ** (17548112), Morf4l2 * (17544754), Morf4l2 **(17548112), Rpl32 *(17469936), Rpl32 **(17549432), Sema4d * (17292562), Sema4d ** (17292569).

Finally, to identify potential interactions between age-related mRNA previously described (Supplementary Table 1) [5] and the changes of miRNA expression observed in this study, we determined whether the predicted target genes of the age-modified miRNAs were either high or low expressed with age compared to all expressed genes (Supplementary Table 9). Accordingly, we generate a heatmap of selected altered expressed miRNAs targets, based on their relevance and relation to the enriched pathways observed in old mice lungs (Figure 8A–8C). For example, the miR-30 family has been implicated in autophagy, apoptosis, oxidative stress, and inflammation [13]. On the other hand, miR-23b and miRNA 29c target extracellular matrix-related genes that also change with aging [14–16].

## DISCUSSION

Lung aging is associated with structural remodeling of airways and alveolar-capillary units leading to a progressive reduction in respiratory function and a higher susceptibility to developing chronic diseases [2, 3].

We recently examined the transcriptomic profile of the lungs of young and old normal and Zmpste24 deficient mice after bleomycin-induced lung fibrosis to explore some of the fibrotic mechanisms associated with accelerated aging [5]. Regarding normal mice, we found 575 genes differentially expressed in old WT mice compared to the young ones (390 upregulated and 185 downregulated). In the present study, we aimed to re-analyze these data to identify pathways and biological processes associated with physiological aging.

Our findings through functional enrichment analyses using Ingenuity Pathway Analysis revealed inflammation, senescence, and apoptosis as some of the most enriched signaling pathways in aged lungs. Concerning inflammation, the most upregulated gene was TREM-1, a receptor of the innate immune system, expressed primarily by myeloid cells and associated with proinflammatory responses [17]. Likewise, toll-like receptor signaling (TLR) was also upregulated. Importantly, the magnitude of the inflammatory response rests on the activation of pattern recognition receptors, which critically includes the family of TLR, which are also implicated in the innate immunity during infectious and non-infectious disorders [18]. Accordingly, NLRP3 (NOD-, LRR- and pyrin domain-containing protein 3), an intracellular sensor, and key component of the inflammasome was also upregulated in the lungs from old mice [19]. The inflammasome represents a group of cytosolic multiprotein signaling complexes responsible for the activation of inflammatory responses [20]. Interestingly, Ifi-16, a member of the HIN-200 (hematopoietic interferon-inducible nuclear antigens with 200 amino acid repeats), was the most over-expressed transcription regulator in old lungs. IFI16 is a nuclear protein involved in the regulation of virus sensing and virus restriction released to extracellular matrix upon injury or stress conditions, recently, new findings demonstrated that IFI16 acts as a damage-associated molecular pattern (DAMP), triggering inflammation through Toll-like receptor 4 (TLR4) activation [21]. Moreover, Ifi-16 deficiency led to a severely attenuated type I IFN response to DNA ligands and viruses [22]. Likewise, Ifi-16/cGAS interaction is required for Stimulator of Interferon Genes (STING), a key player in host defense against pathogens, activation after DNA stimulation [23].

These findings support the notion that inflammaging, the chronic, low-grade inflammation that develops with age, is a common process of normal lung aging and may contribute to susceptibility to age-related pathologies [24], including DNA lesion-induced carcinogenesis [25]. Actually, a recent published systematic review and meta-analysis provides epidemiological data on the relationship between chronic inflammation and cancer incidence including lung cancer [26]. Interestingly, IPA analysis performed in our data showed “Invasion of tumor” as one of the pathways affected by aging; some of the dysregulated genes were *AREG, BIRC5, CCL2, CD14, CTSB, FN1, HMOX1, IL6, MMP14, MMP3, PLAU, S100A8, SERPINE1, SPP1, THBS1, VCAN*.

Cytokines are a major component of the biology of aging and inflammaging, and specifically, increased levels of interleukin (IL)-1β, IL-6, and tumor necrosis factor (TNF) have been observed in aging rodent and human studies [27]. In our study, several of them, such as IL-6 and TNF, were upregulated in the old lungs.

Importantly, lung inflammation during aging can be associated with senescence, that figured among the most enriched canonical pathways in the lung during aging. Senescence is characterized by a stable cell cycle arrest and by the secretion of a variety of cytokines and other soluble factors with pleiotropic functions known as SASP [28]. We found that both, CDKN1a (a cell cycle regulator) and IL6 (a critical SASP mediator), were upregulated in the aged lung. Under physiological conditions, senescence is activated as a repair response to damage or as an antineoplastic process. However, during aging there is an increase of senescent cells while the rate of their removal decreases resulting in their accumulation contributing with disrepair.

Finally, the putative role of the lung microbiome in the observed inflammatory signals is unclear. Evidence has shown the loss of diversity of lung microbiota with increasing age together with age-dysregulation of the immune response and other factors plausibly contributes to the process of inflammaging [29, 30].

The evaluation of the molecular and cellular functions category also revealed apoptosis of several lung cell populations as a major enriched pathway. The machinery of apoptosis is complex, involves many signaling pathways, and can be triggered through either the caspase-mediated extrinsic or intrinsic pathways [31]. Actually, several aging hallmarks may increase susceptibility to programmed cell death, including genomic instability, mitochondrial dysfunction, oxidative stress, and telomere attrition. Additionally, the ineffective clearance of apoptotic bodies by neighboring phagocytes may worsen lung integrity, and it is well known that the capacity of macrophage phagocytosis of apoptotic cells is diminished with age which contributes to an impairment of the resolution of inflammation with increasing age [32]. Our findings indicate that the programmed cell death mechanisms become activated with aging triggering loss of structural cells (e.g., epithelium, endothelium as found in this study) with the consequent decline of lung function. Recent data show that that similar pathways found in this study, are dysregulated in lung epithelial cells [33].

MicroRNAs are key post-transcriptional regulators of gene expression implicated in numerous biological processes, including cell growth, proliferation, differentiation, and organismal metabolism and development [34]. These non-coding miRNAs have been increasingly recognized as relevant regulators of aging. However, although important advances have been performed regarding the functional roles of miRNAs in several organs during healthy mammalian aging, studies in the lungs are scanty [35].

Our findings showed that 25 miRNAs were significantly different in the lungs of old mice, eight upregulated and 17 downregulated, compared with their young lungs. Using miRNet database we examined the putative participation of these miRNAs in the control of the dysregulated pathways that we observed during lung aging. We identified TNFα, mTOR, TGFβ, WNT, FoxO, Apoptosis, Cell cycle, and p53 signaling pathways as the potential targets of let-7b, let7c, miR29b, miR181b, miR147a, and miR23. Cell cycle and p53 signaling pathway are key processes in cellular senescence [36, 37], a hallmark of aging, and TNFα, mTOR, TGF-β, and WNT signaling pathway have been involved in the regulation of apoptosis, inflammation, and fibrosis [38, 39]. Likewise, the downregulation of let7b/c has been found in senescent cells [40]. Altogether, the target of the dysregulated miRNAs found in this study may promote in old lungs susceptibility to damage, inflammation, and fibrosis [41]. Finally, enriched processes regulated by miR-23-3p, miR-29b-3p, and miR-30d-5p also include regulation of inflammation and cell death processes correlating with the global changes revealed in the transcriptome of old lungs generated by IPA. The relevance of the members of the miR30 family in the regulation of apoptosis, oxidative stress, and inflammation in different organs has been previously reported [13]. For instance, downregulation of miR30 levels were associated with podocyte apoptosis and altered activation of the p38 MAPK signaling. In the lung, we found that during aging miR30 was downregulated and the most enriched GO pathways were cell death, supporting the importance of miR30 in the regulation of apoptosis.

In summary, our findings indicate that during aging, dysregulation of several miRNAs and its target genes are involved in alterations of cellular metabolic processes, induction of apoptosis, senescence, and the development of a pro-inflammatory phenotype.

## MATERIALS AND METHODS

### Animals

Young (1-month-old) and old (18-month-old) C57BL/6 wild type mice were housed in specific pathogen-free conditions. The Ethics Committee of the National Institute of Respiratory Diseases of Mexico (INER) approved all experiments.

### RNA extraction and preparation

RNA was extracted using TRIzol reagent (Life Technologies, Grand Island, New York, NY, USA) following the manufacturers' instructions. Purity and efficiency were verified by spectrophotometry (NanoDrop, Wilmington, DE, USA) and bioanalysis (Agilent, Palo Alto, CA, USA).

### Microarray analysis of differential gene expression

Microarray data can be downloaded from GEO database (GSE123293) [5]. The biotin-labeled cRNA was purified, fragmented, and hybridized to GeneChip™ Mouse Gene 2.0 ST Array (Affymetrix®). For each group, three different biological samples were used. The microarray data was analyzed by R software version 4.0.2 (http://www.r-project.org/) [42] and Bioconductor version 3.11 (http://www.bioconductor.org/) [43]. To identify significant differences between gene expression in each condition, all data were analyzed by Limma package version 3.44.4 using a linear model based on Bayes empirical method [44]. Representative data were considered significantly with higher *p*-values (adjusted *p*-value < 0.05).

### Interactome/network analysis of differentially expressed genes using IPA software

Significantly differentially expressed genes were subjected to a comprehensive exploration to identify their biological functions. Gene interaction networks, bio functions, and pathway analysis were generated by Ingenuity Pathway Analysis (IPA) (Ingenuity Systems; Mountain View, CA, USA, v2021), using as reference set the Mouse 2.0 ST Array, and consider only relationships where confidence = Experimentally Observed. Differentially expressed genes were mapped to genetic networks available in the IPA software and were then ranked by score. The significance was set at a *p*-value of 0.05, and logFC cutoff of ≥1,1≤.

### microRNAs enrichment analysis

All miRNAs were selected from the list of differentially expressed genes according to its p-value < 0.05. We built a network using miRNet v.2.0 (http://mirnet.ca, last update 2021-01-29) [12, 45], that contains miRNAs-target interactions from miRTarBase v8.0 [46], TarBase v8.0 [47] and miRecords [48]. Through the platform, we performed enrichment analysis using KEGG and Gene Ontology Biological Process. Besides, we used package multiMiR version 1.10.0 [49–51], to select validated target genes for the miRNAs. Target genes were looked through microarray data, and depending on the miRNA level expression, we selected those target genes that were congruent with the expected effect (miRNA up -> target gene down; miRNA down -> target gene expressed).

### Statistical analysis

Statistical differences for two groups were analyzed by Student’s t-test. Results are expressed as mean ± SD. or S.E.M., and *p*-value < 0.05 was considered statistically significant.

### Visualization

Package ggplot2 version 3.3.2 was used to plot most of the bar chart figures [52]. To build the heatmaps we used pheatmap package version 1.0.12 [53].

## Supplementary Material

Supplementary Figures

Supplementary Table 1

Supplementary Table 2

Supplementary Table 3

Supplementary Table 4

Supplementary Table 5

Supplementary Table 6

Supplementary Table 7

Supplementary Table 8

Supplementary Table 9
