# Supplementary material for: Transcriptomic profile of the mice aging lung is associated with inflammation and apoptosis as important pathways
Source: Aging (Albany NY). 2021 May 12;13(9):12378–94. doi: 10.18632/aging.203039 (PMC8148450; doi:10.18632/aging.203039)
Supplement: Supplementary Figures [file aging-13-203039-s001.pdf]

SUPPLEMENTARY FIGURES

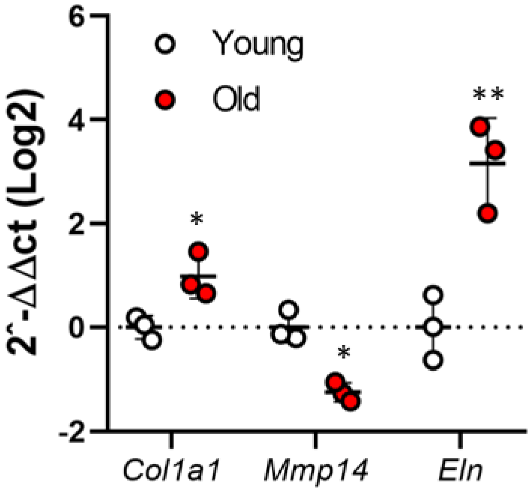

Supplementary Figure 1. Dysregulated genes during aging. *Col1a1, Mmp14,* and *Eln* mRNA expression were validated by quantitative RT-PCR. Data shows ratio fold-change of selected genes in the lung reported as log2 ratio between old mice compared to young ones. Genes were normalized to 18S rRNA. Graph is expressed in mean ± SEM where each dot represents a different individual(n=3); *p< 0.05, **p< 0.01.

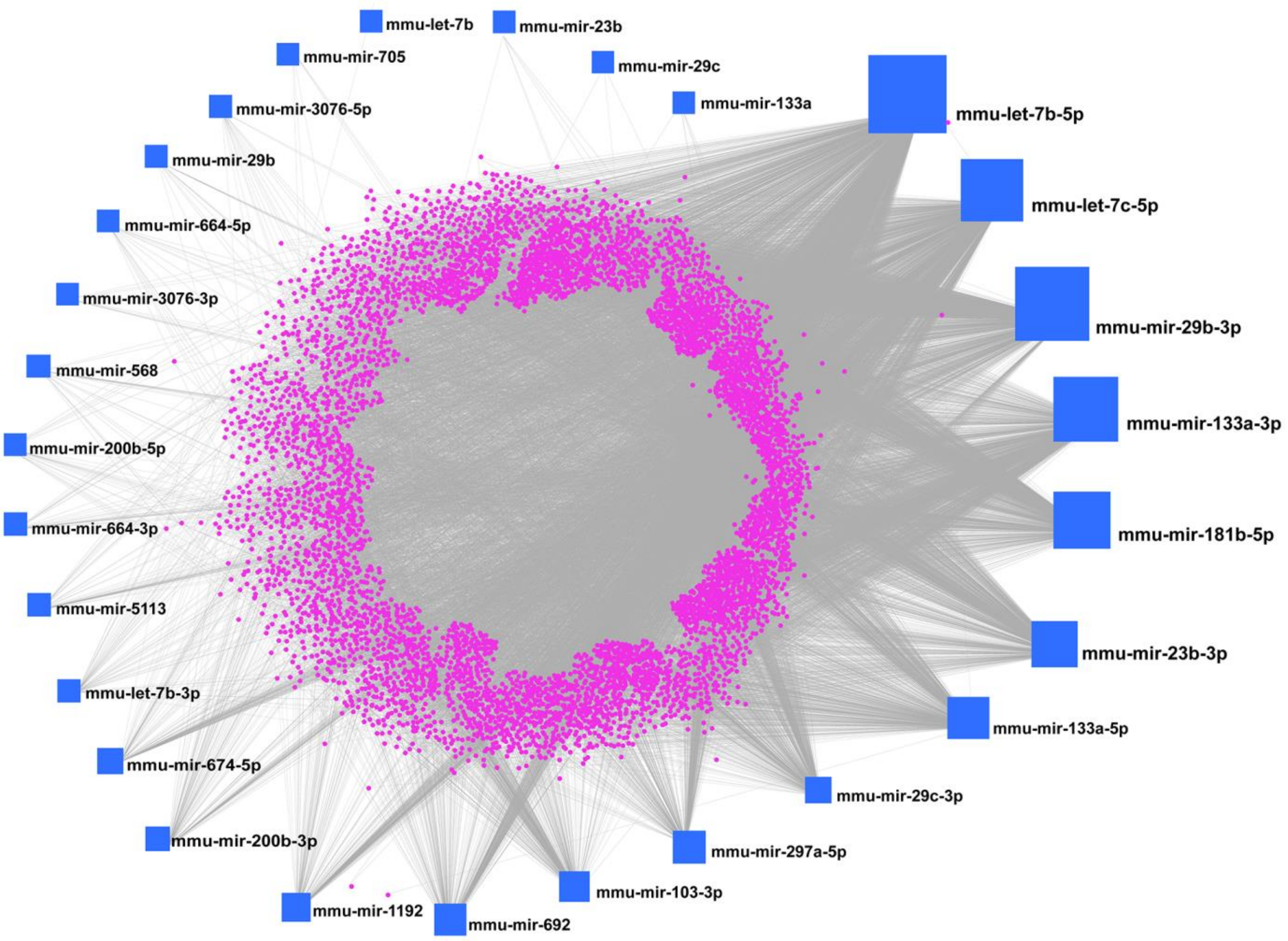

Supplementary Figure 2. miRNA network associated with changes in the lung during aging. MiRNet network is composed of miRNAs differentially expressed in old versus young and their target genes. miRNAs are represented as blue squares and the size represents the number of target genes (pink points). The total of target genes is 6,158, and there are 12,450 edges.
