# Supplementary material for: Transcriptomic profile of the mice aging lung is associated with inflammation and apoptosis as important pathways
Source: Aging (Albany NY). 2021 May 12;13(9):12378–94. doi: 10.18632/aging.203039 (PMC8148450; doi:10.18632/aging.203039)
Supplement: Supplementary Table 5 [file aging-13-203039-s006.pdf]

Supplementary Table 5. Dysregulated microRNAs.

PROBEID	ENSEMBL	SYMBOL	GENENAME	logFC	adj.P.Val
17220526	ENSMUSG00000105770	Mir664	microRNA 664	1.8020447	0.047214958
17375503	ENSMUSG00000076309	Mir147	microRNA 147	1.6602874	0.012075155
17380764	ENSMUSG00000065460	Mir133a-2	microRNA 133a-2	0.967884	0.023524216
17549192	ENSMUSG00000076012	Mir705	microRNA 705	0.6970218	0.031233682
17288650	ENSMUSG00000094403	Mir692-2	microRNA 692-2	0.6296001	0.022178446
17288650	ENSMUSG00000076033	Mir692-3	microRNA 692-3	0.6296001	0.022178446
17316977	ENSMUSG00000084458	Mir1907	microRNA 1907	0.6053362	0.047787288
17298183	ENSMUSG00000093156	Mir3076	microRNA 3076	0.5010683	0.026117076
17270974	ENSMUSG00000093273	Mir3064	microRNA 3064	-0.5050524	0.026829734
17374468	ENSMUSG00000076376	Mir674	microRNA 674	-0.5464373	0.046645879
17313169	ENSMUSG00000092868	Mir5113	microRNA 5113	-0.6024039	0.014793273
17267184	ENSMUSG00000093278	Mir5110	microRNA 5110	-0.6529927	0.036771377
17276396	ENSMUSG00000092922	Mir5101	microRNA 5101	-0.6665453	0.003986028
17221026	ENSMUSG00000065412	Mir29b-2	microRNA 29b-2	-0.8026317	0.041822403
17221026	ENSMUSG00000065548	Mir29c	microRNA 29c	-0.8026317	0.041822403
17313860	ENSMUSG00000065564	Mirlet7b	microRNA let7b	-0.8184671	0.041335966
17376508	ENSMUSG00000065563	Mir103-2	microRNA 103-2	-0.8336789	0.036771377
17358219	ENSMUSG00000080626	Mir1192	microRNA 1192	-0.9261903	0.007197001
17317708	ENSMUSG00000065437	Mir30d	microRNA 30d	-0.9626485	0.004933634
17325723	ENSMUSG00000076372	Mir568	microRNA 568	-1.0469047	0.020377963
17288105	ENSMUSG00000065599	Mir23b	microRNA 23b	-1.0660549	0.019866322
17433975	ENSMUSG00000065549	Mir200b	microRNA 200b	-1.1705651	0.001692767
17217846	ENSMUSG00000065458	Mir181b-1	microRNA 181b-1	-1.1826916	0.037944573
17366734	ENSMUSG00000076983	Mir297a-2	microRNA 297a-2	-1.2654825	0.030506634
17313858	ENSMUSG00000065608	Mirlet7c-2	microRNA let7c-2	-1.477739	0.002349259
